# Exploration of the Fire-Retardant Potential of Microencapsulated Ammonium Polyphosphate in Epoxy Vitrimer Containing Dynamic Disulfide Bonds

**DOI:** 10.3390/polym15132839

**Published:** 2023-06-27

**Authors:** Wenlong Shao, Tongbing Li, Fei Xiao, Fubin Luo, Yong Qiu, Yanyan Liu, Bihe Yuan, Kaiyuan Li

**Affiliations:** 1 School of Safety Science and Emergency Management, Wuhan University of Technology, Wuhan 430070, China; shaowenlong@whut.edu.cn (W.S.); yanyan.liu@whut.edu.cn (Y.L.); bhyuan@whut.edu.cn (B.Y.);; 2 Guangdong Advanced Thermoplastic Polymer Technology Co., Ltd., Dongguan 523125, China; rd03@atpchem.com; 3 Engineering Research Center of Polymer Green Recycling of Ministry of Education, College of Environmental and Resource Sciences, Fujian Normal University, Fuzhou 350007, China; 4 China Light Industry Engineering Technology Research Center of Advanced Flame Retardants, Beijing Technology and Business University, Beijing 100048, China; yongqiu@btbu.edu.cn

**Keywords:** fire retardant, epoxy vitrimers, thermal stability, microencapsulated ammonium polyphosphate

## Abstract

Epoxy vitrimers appear as a promising alternative to common epoxy thermoset composites. Nevertheless, the possibilities of applying these materials are limited due to their high flammability which may cause high fire risks. To date, the flame-retardant epoxy vitrimer systems reported in the literature almost all rely on intrinsic flame retardancy to achieve high fire safety; however, the complex and expensive synthesis process hinders their large-scale application. In this work, disulfide-based epoxy vitrimer (EPV) was fabricated with 4, 4′-dithiodianiline as the curing agent, and microencapsulated ammonium polyphosphate (MFAPP) was employed as a potential additive flame retardant to improve their fire retardancy. As a comparative study, common epoxy (EP) composites were also prepared using 4,4′-diaminodiphenylmethane as the curing agent. The results showed that the introduction of dynamic disulfide bonds led to a reduction in the initial thermal decomposition temperature of EPV by around 70 °C compared to EP. Moreover, the addition of 7.5 wt.% of MFAPP endowed EP with excellent fire performance: the LOI value was as high as 29.9% and the V-0 rating was achieved in the UL-94 test (3.2 mm). However, under the same loading, although EPV/MFAPP7.5% showed obvious anti-dripping performance, it did not reach any rating in the UL-94 test. The flame-retardant mechanisms in the condensed phase were evaluated using SEM-EDS, XPS, and Raman spectroscopy. The results showed that the residue of EPV/MFAPP7.5% presented numerous holes during burning, which failed to form a continuous and dense char layer as a physical barrier resulting in relatively poor flame retardancy compared to EP/MFAPP7.5%.

## 1. Introduction

Epoxy vitrimers are constantly emerging. They are a special class of polymers that integrate the unique characteristics of both thermoplastics and thermosets [1,2,3,4]. The presence of abundant dynamic exchangeable bonds in their structure endows epoxy vitrimers with high self-healing (reparable) [5,6], recyclable [7,8], and reprocessing [9,10] capabilities, showing the potential to extend their lifetime and reduce maintenance costs in many application fields [11]. While epoxy vitrimers have been developed in recent years, the main bottleneck persists in their intrinsic flammability [12], which releases a large amount of heat and smoke during burning, resulting in high fire risk during their application. 

To address this issue, efforts have been made to explore the fire retardancy of epoxy vitrimers. Most studies focused on the incorporation of flame-retardant elements into cross-linked EP networks. In this regard, some phosphorus-/nitrogen-containing functional groups, such as 9,10-dihydro-9-oxa-10-phosphazene-10-oxide (DOPO) derivatives [13,14], 2-(bis(2-hydroxyethyl)amino)ethyl diphenylphosphinate [15], cyclolinear cyclotriphosphazene [16], cyclophophazene [17,18], and β-ketoester-containing phosphonate [19], were introduced into the crosslinked network of EP by modifying epoxy monomers or curing agents. For instance, Chen et al. prepared intrinsically flame-retardant epoxy vitrimers by introducing dynamic phosphorus-containing ester linkages into the crosslinking networks. The resulting epoxy vitrimers exhibited desirable thermal stability and excellent fire retardancy with a V-0 rating in the UL-94 test (3.2 mm) [13]. Intrinsic flame retardants are connected to the EP matrix through chemical bonds; they participate in curing and become a component of the cured system structure [20], ensuring the uniformity of EP vitrimers and preventing the precipitation of flame retardants [21]. However, this inevitably increases the complexity of the synthesis and curing process of epoxy vitrimers which hinders its commercial application. Comparatively, additive flame retardants do not participate in the curing reaction and are the most ideal industrial solution due to their relative cheapness and convenience [22,23,24]. Moreover, inspired by the recycling of carbon fibers in epoxy vitrimers [25], additive flame retardants and epoxy monomers are expected to be recycled separately. 

In this work, a common epoxy vitrimer (EPV) was prepared based on the exchangeable disulfide bonds using bisphenol A diglycidyl ether as the epoxy monomer and 4, 4′-dithiodianiline (DTDA) as the curing agent [26]. To endow the EPV with flame retardancy, melamine–formaldehyde resin microencapsulated ammonium polyphosphate (MFAPP) was incorporated as a promising flame retardant due to the excellent compatibility, flame retardancy, and water resistance, benefiting from the presence of the organic MF coating on the surface of APP [27,28,29]. As a comparative study, the fire performance of epoxy composites was also studied with 4,4’-methylenedianiline (DDM) as the curing agent and MFAPP as the flame retardant. Overall, the details of fire behaviors, flame retardancy, and the mechanism of actions of flame-retardant EP and EPV composites were investigated to assess the flame-retardant potential of MFAPP for epoxy vitrimers.

## 2. Experimental Setup

### 2.1. Materials

Diglycidyl ether of bisphenol-A (DGEBA, E-51, WSR618) with an epoxide equivalent weight of 184–200 g/eq was supplied by Nantong Xingchen Synthetic Material Co., Ltd., Nantong, China. 4, 4′-Diaminodiphenylmethane (DDM, 99%) and 4, 4′-dithiodianiline (DTDA, 98%), supplied by Shanghai Aladdin Biochemical Technology Co., Ltd., Shanghai, China, were used as the curing agents for the curing of the epoxy matrix. Melamine–formaldehyde resin microencapsulated ammonium polyphosphate (MFAPP, TF-MF201, ammonium polyphosphate with crystalline form II) was purchased from Guangzhou Zhanpu Chemical Co., Ltd., Guangzhou, China.

### 2.2. Preparation of the Cured Epoxy Composites

For epoxy resin (EP) composites, DGEBA was added to a beaker with magnetic stirring at 95 °C for 15 min. Subsequently, various contents of MFAPP were slowly added into DGEBA and adequately mixed for 20 min to form a uniform mixed system. Then, DDM was incorporated into the mixture and continuously stirred for another 20 min. After degassing at 105 °C for 5 min, the blend was poured into a pre-heated mold and cured at 120 °C for 2 h and then post-cured at 150 °C for 2 h. Finally, the cured specimens were naturally cooled to room temperature. Epoxy vitrimer (EPV) composites were fabricated by the same method, except that 4, 4′-dithiodianiline was used as a curing agent. Moreover, the pre-curing time was increased to 40 min to avoid the precipitation of flame retardants. The detailed formulas are presented in Table 1.

### 2.3. Characterization

The thermogravimetric analysis (TGA) was performed to evaluate the thermal stability of the samples using a simultaneous thermogravimetric analyzer (NETZSCH STA 449 F3, NETZSCH, Bayern, Germany). Approximately 5 mg of samples were heated from 25 °C to 800 °C at a heating rate of 10 °C/min under nitrogen atmospheres. Limiting oxygen index (LOI) values of the samples were measured using an AOI LOI apparatus (Motis Fire Technology Co., Ltd., Kunshan, China) standardized as ASTM D2863 with dimensions of 130 × 7.0 × 3.2 mm^3^. The UL-94 vertical burning test was carried out using an M607 horizontal-vertical combustion apparatus (Qingdao Shang-fang Instrument Co., Ltd., Qingdao, China) according to ASTM D3801-2020 standards. The dimensions of the samples were 127 × 12.7 × 3.2 mm^3^. A Thermo Scientific K-Alpha X-ray photoelectron spectrometer (XPS) (Thermo Fisher Scientific, Waltham, MA, USA) was used to determine the elemental composition and oxidation states of elements at the surface of the residues after the UL-94 test, and Al Ka radiation (1361 eV) was used as the excitation source. The LabRAM Odyssey high-speed and high-resolution confocal microscopic Raman spectrometer (HORIBA, Gières, France) was applied to investigate the graphitization degree of the char residue. The Raman spectra of residual chars were obtained at room temperature under the excitation line of a 532 nm laser. The morphology and element mappings of char residue after the UL-94 test were performed using a Gemini 300 scanning electron microscope (SEM, ZEISS, Oberkochen, Germany), which was integrated with an energy-dispersive X-ray (EDX) microanalyzer for elemental analysis.

## 3. Results and Discussion

### 3.1. Thermal Stability

**Figure 1:**
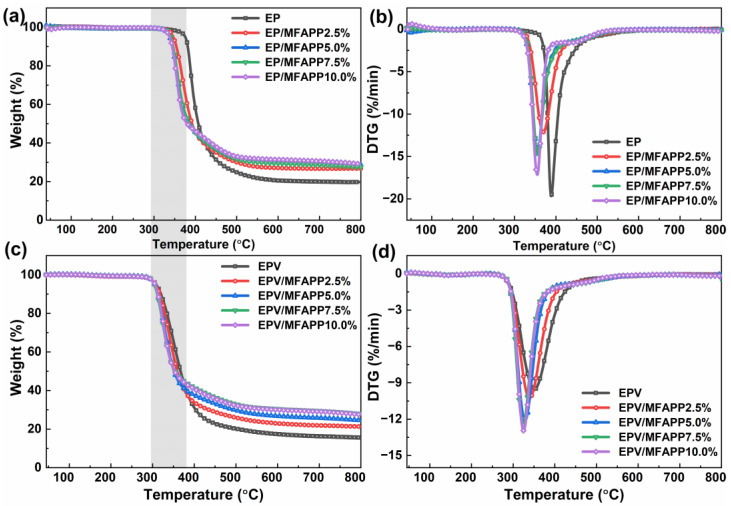
TGA and DTG curves of EP (**a**,**b**) and EPV composites (**c**,**d**).

TGA was performed to investigate the thermal performance of EP and EPV composites under a nitrogen atmosphere, and the TGA/DTG curves and the relative data are presented in Figure 1 and Table 2. It is clear that the incorporation of MFAPP into the epoxy matrix inevitably affects the thermal stability of EP and EPV composites. For EP composites, it is evidently observed from DTG curves that a neat EP displays one-step degradation behavior as well as EP/MFAPP composites, occurring approximately from 300 to 500 °C. The initial decomposition temperature (T_5%_) of neat EP is 375 °C, and the thermal degradation rate rapidly reaches the maximum at 388 °C (T_max_). There is a 19.7 wt.% residue formed at 800 °C in the EP matrix, indicating the presence of abundant aromatic/polyaromatic structures. It is worth noting that with the increase in MFAPP loading (2.5–10.0 wt.%), the T_5%_ values of EP composites with MFAPP gradually decrease and stabilize at around 340 °C, and the char residues gradually increase to approximately 29 %. This phenomenon is attributed to the weak bonds of P-O-C and P-N in the structure of MFAPP [30] and is probably ascribed to the decreased cross-linking density of EP due to the addition of MFAPP [31]. The TGA and DTG curves of EPV composites exhibit a similar behavior to those of EP composites. Nevertheless, there is a remarkable reduction (~67 °C) in the T_5%_ for EPV compared to EP, which might be attributed to the instability of disulfide bonds in the crosslinked networks [2]. The incorporation of MFAPP does not significantly reduce the thermal stability (T_5%_ and T_max_) of EPV, and char residues at 800 °C increase with the increase in MFAPP loading. In conclusion, although the incorporation of MFAPP leads to a certain reduction in the thermal stability of EP and EPV composites, the char yields of those composites are significantly improved, which is beneficial to the flame retardancy of EP. 

### 3.2. Flame Retardancy

**Figure 2:**
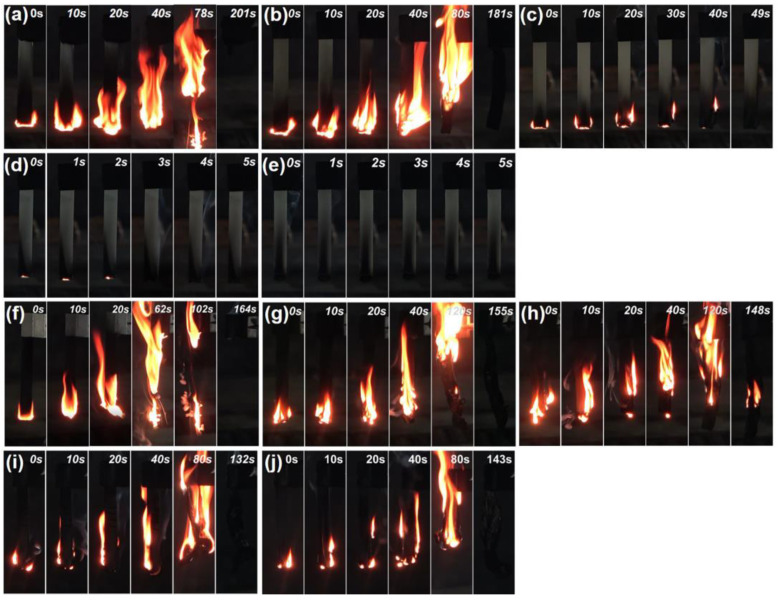
Fire behaviors of (**a**–**e**) EP and (**f**–**j**) EPV composites after the first ignition (0, 2.5 wt.%, 5 wt.%, 7.5 wt.%, and 10 wt.% MFAPP loading).

**Figure 3:**
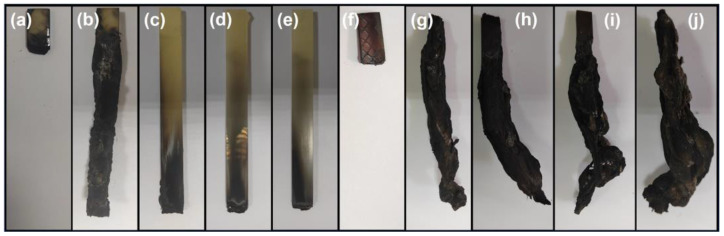
Residues of (**a**–**e**) EP and (**f**–**j**) EPV composites after the UL-94 tests (0, 2.5 wt.%, 5.0 wt.%, 7.5 wt.%, and 10.0 wt.% MFAPP loading).

The flammability, fire retardancy, and anti-dripping behavior of EP and EPV composites were evaluated using LOI and UL-94 vertical burning tests [32,33,34], and the corresponding results are presented in Table 3 and Figure 2 and Figure 3. It is seen that the LOI value of EP is only 23.4%, and it burns violently after ignition with severe dripping fire behavior, which is considered as a significant fire hazard [35]. With the incorporation of MFAPP at a low loading (2.5%), EP/MFAPP2.5% still burns out (no rating) in the UL-94 test but no dripping occurred (Figure 2). By further increasing the loading of MFAPP to 5.0 wt.%, 7.5 wt.%, and 10.0 wt.%, the flame retardancy of EP/MFAPP composites gradually improved, and when the loading reached 7.5 wt.%, a V-0 rating was achieved for EP/MFAPP7.5% with an LOI value of 29.7%.

Regarding EPV composites, neat EPV is very flammable, showing an LOI value of 19.9% and no rating (NR) in the UL-94 test. As the loading of MFAPP increases to 10 wt.%, the LOI value gradually increases to 25.3%, which is far lower than the EP composite with the same content (Table 3). However, in terms of the UL-94 ratings, EPV composites showed poor results relative to the EP composites (Figure 2f–j). Although all EPV composites exhibited significant enhancements in anti-dripping behavior, the UL-94 ratings of EPV/MFAPP systems seemed to not largely improve with the increasing loading of MFAPP, as all EPV composites failed to pass any rating in the UL-94 test. It can be concluded that the addition of MFAPP does not significantly improve the flame retardancy of EPV.

Figure 3 presents the char residues of EP and EPV composites after UL-94 tests. The residual chars of neat EP and EPV almost burn out, which illustrates the weak char formation capacity and high fire risk of neat EP and EPV matrices. It is observed that increasing the content of MFAPP causes the EP composites to self-extinguish over a shorter burning distance, resulting in the formation of a dense carbon layer. On the other hand, when MFAPP is introduced into the EPV matrix, the yields of char residues for the EPV composites rise significantly; however, all EPV composites do not extinguish spontaneously within 60 s after the first ignition. Visual observation shows that intumescent char residues are formed due to the presence of MFAPP, which does not retard the burning of the matrix underneath. 

### 3.3. Morphology and Chemical Structure of Char Residues

#### 3.3.1. SEM-EDS Analysis

On the basis of the above analysis of LOI and UL-94 tests, it can be concluded that EP/MFAPP composites possess better flame retardancy than EPV/MFAPP composites under the same loading. To further investigate its relevant mechanisms in the condensed phase, the micro-morphologies of residual chars of EP/MFAPP7.5% and EPV/MFAPP7.5% after the UL-94 test were analyzed by SEM-EDS. 

**Figure 4:**
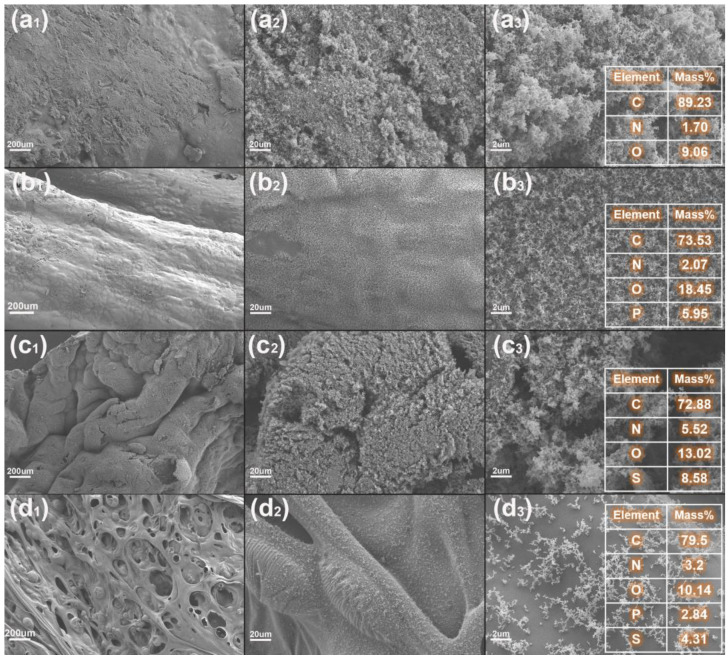
SEM-EDS images of the exterior surface of the char residues after the UL-94 test: (**a_1_–a_3_**) neat EP, (**b_1_–b_3_**) EP/FMAPP7.5%, (**c_1_**–**c_3_**) neat EPV, (**d_1_–d_3_**) EPV/FMAPP7.5%.

It is observed in Figure 4a,c that the surface residues of neat EP and EPV exhibit very loose and porous morphologies. In contrast, due to the addition of MFAPP, the surface of char residues of EP/MFAPP7.5% become compact, smooth, and homogeneous, implying the improvement of the char quality (Figure 4c) [36]. The continuous and dense char layer serves as an effective physical barrier to inhibit the transfer of heat and prevent the internal pyrolysis products from being released into the combustion zone [37]. For EPV/MFAPP7.5%, there are numerous holes observed on the surface of char residues (Figure 4d). These holes allow combustible pyrolysis products to be released from the internal matrix into the external environment, resulting in poor flame retardancy.

EDS analysis was performed to study the chemical composition of the char residues, as shown in Figure 4. In the EDS spectrum of EP/MFAPP7.5%, the main composition of the residue is carbon (73.53%), oxygen (18.45%), nitrogen (2.07%), and phosphorus (5.96%). The relatively high phosphorus content mainly comes from the phosphorus-containing species formed by the decomposition of MFAPP, which are mainly orthophosphoric acid and phosphoric acid, according to previous work [38,39]. Moreover, the oxygen content is also high (18.45 %), implying that the formation of a continuous and compact char layer is related to the increase in phosphorus and oxygen content [31]. It is concluded that the introduction of MFAPP in the EP matrix promotes the formation of a char layer with a more phosphorus-crosslinked structure, improving the quality of the char layer. For the EDS spectrum of EPV/MFAPP7.5%, it is found that the phosphorus content (2.84%) on the char layer from the char residue is much lower than that of the EP/MFAPP7.5%. Sulfur emerges in the char residue of EPV/MFAPP7.5% due to the presence of DTDA as the curing agent. 

#### 3.3.2. Elemental Mappings

**Figure 5:**
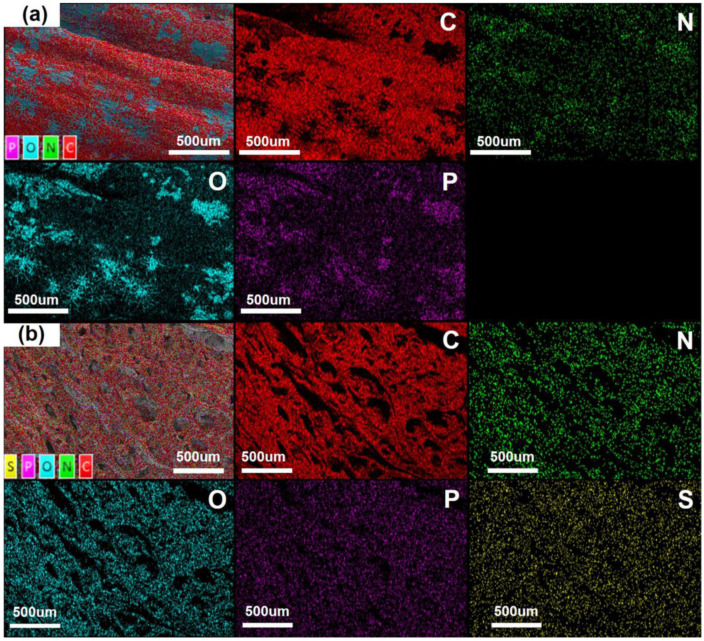
EDS mapping images of total elements, C, N, O, P, and S elements of the exterior char residues after the UL-94 tests: (**a**) EP/FMAPP7.5%, (**b**) EPV/FMAPP7.5%.

The distribution of species in the residual chars is very important when explaining the mechanism in the condensed phase. Figure 5a shows the elemental mapping images of C, O, N, and P elements, respectively, of the surface char of EP/MFAPP7.5%. The results provide visible evidence that MFAPP decomposes to form phosphorus-containing species, which combine with oxygen (see the O and P elemental mappings in Figure 5) to form phosphoric acid/orthophosphates/pyrophosphates and the related analogues during burning. The phosphorus-containing species derived from MFAPP promote the dehydration and carbonization of the epoxy matrix and form a continuous and dense carbonaceous char layer. It is found that the aggregated phosphate species are embedded on the surface of the carbon layer, which enhances the cohesion and resistance of the char residues, thereby improving its flame retardancy. For EPV/MFAPP7.5%, the mapping images confirm the presence and dispersion of C, O, N, P, and S elements, which indicates those elements are uniformly distributed on the surface of char. 

#### 3.3.3. XPS Analysis

**Figure 6:**
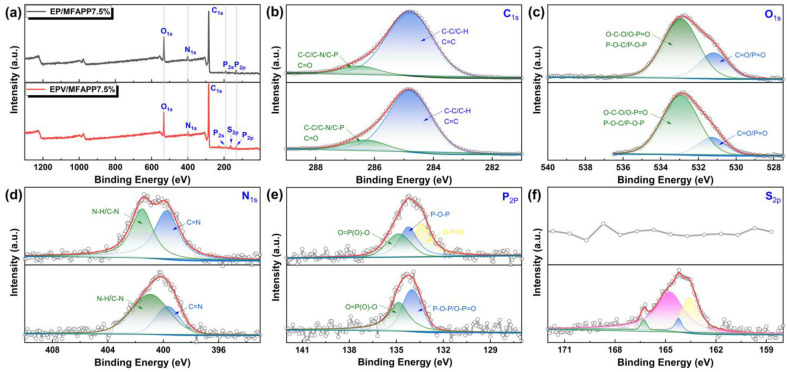
(**a**) XPS survey spectra and high-resolution XPS spectra of (**b**) C_1s_, (**c**) O_1s_, (**d**) N_1s_, (**e**) P_2s_, and (**f**) S_2s_ of the exterior char residues of EP/FMAPP7.5% and EPV/FMAPP7.5% after the UL-94 tests.

To further explore the effect of MFAPP on the charring of EP and EPV, XPS analysis was conducted for the chars of EP/MFAPP7.5% and EPV/MFAPP7.5%. Figure 6 shows the C_1s_, O_1s_, N_1s_, P_2p_ and S_2p_ spectra of the external char of EP/MFAPP7.5% and EPV/MFAPP7.5%. In the C_1s_ spectra, the peak at 284.8 eV is assigned mainly to the C-H, C-C, and C=C in the aliphatic and aromatic species in char, while the peak at 286.5 eV corresponds to C-O, C-N, C-P, and C=O linkages [40]. The high-resolution O_1s_ spectra consist of two characteristic peaks corresponding to the double-bonded oxygen (C=O and P=O groups) in phosphate and carbonyl compounds at a binding energy of 531.3 eV and single-bonded oxygen (-O-) in O-C-O, C-O-P, O-P=O, and P-O-P at around 533.0 eV [41]. In addition, in the N_1s_ spectrum of EP/MFAPP7.5% (Figure 6), the broad bands centered at 399.8 and 401.5 eV are assigned to C=N group and C-N/N-H groups, respectively, while the peak intensity at 401.0 eV for EPV/MFAPP7.5% decreases compared to EP/MFAPP7.5%, indicating that less nitrogen-containing aromatic heterocyclic cross-linking structures are formed in the char residue. The deconvoluted P_2p_ region spectrum of EPV/MFAPP7.5% shows two signals at 134.0 eV attributed to O-P-C, O-P-O, O-P=O groups formed during the decomposition of MFAPP, while the peak at 134.8 eV is assigned to the structure of pyrophosphates and metaphosphates in P-O-P and PO_3_, respectively [42]. Similar linkages are also found for the P_2p_ survey of EP/MFAPP7.5% char, except that the linkages of O-P=O can be fitted in an independent peak at a binding energy of 133.4 eV. The high-resolution S_2p_ spectrum is detected for EPV/MFAPP7.5% as demonstrated earlier by the EDS results. The signal can be fitted into four peaks, where the binding energies at 163.6 and 164.2 eV are assigned to the S-C and S=C, thiols, or sulfur ethers [43,44], while the peaks observed at 164.8 and 166.3 eV are attributed to the oxidized sulfur species (SO_x_) and sulfoxide sulfur due to the burning [45,46]. Compared to EPV/MFAPP7.5%, the N_1s_ and P_2s_ spectra of EP/MFAPP7.5% showed broader bands, suggesting that a more complex heterocyclic carbonaceous structure containing phosphorus and nitrogen elements was formed. These results verify that the introduction of MFAPP is conducive to the formation of crosslinking structures containing P-O-C, P-O-P, C-N, and C-O-C groups during burning, endowing the resulting char with better barrier effects and stability, effectively insulating the heat and fuel transfer between the gas and condensed phases. 

#### 3.3.4. Raman Analysis

**Figure 7:**
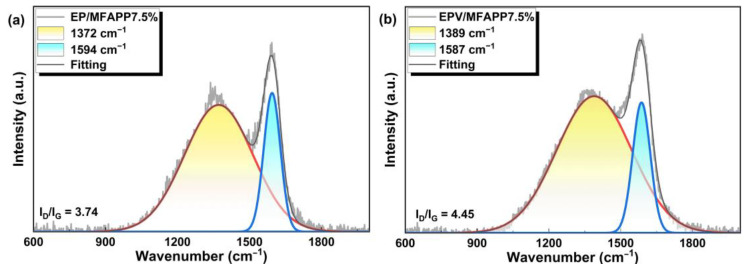
SEM-EDS images of the exterior char residues after the UL-94 tests: (**a**) EP/FMAPP7.5%, (**b**) EPV/FMAPP7.5%.

To investigate the effect of MFAPP on the structure of the char layers of EP and EPV composites, laser Raman spectroscopy was conducted to study the graphitization level of the char of EP/MFAPP7.5% and EPV/MFAPP7.5%, as presented in Figure 7. Two strong characteristic peaks at approximately 1370 and 1590 cm^−1^ are assigned to the D band (disorder band or defect band, indicating the presence of some disorder in the carbon aromatic structure) and G band (tangential vibration mode along the graphitic plane of the tube), respectively. Generally, the ratio of the band intensity of the D to G band (I_D_/I_G_) is an important measure of the order to estimate the graphitization degree of the char residues, and a lower value corresponds to a higher graphitization degree. The I_D_/I_G_ value of EP/MFAPP7.5% is 3.74 and this value increases to 4.45 for EPV/MFAPP7.5%. The phenomenon indicates that compared to EP/MFAPP7.5%, the residue of EPV/MFAPP7.5% presents more defects after burning. It demonstrates that a more graphitized structure in residual char is formed during the burning of EP/MFAPP7.5%, which improves the barrier effect of the heat and fuel transfer. 

### 3.4. Flame-Retardant Mechanisms

**Figure 8:**
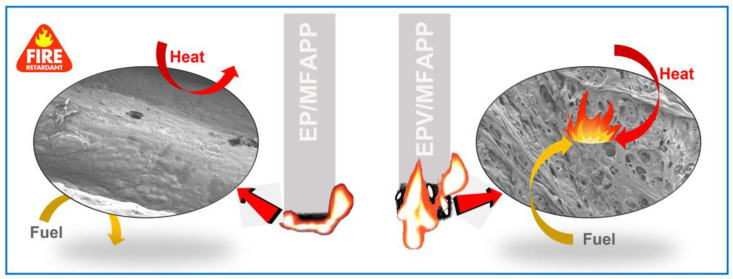
Possible flame-retardant mechanisms of EP/MFAPP and EPV/MFAPP composites.

Based on the above analyses, the possible flame-retardant mechanisms of EP/MFAPP and EPV/MFAPP are proposed as shown in Figure 8. In the condensed phase, phosphonic acid and its derivatives can be formed during the early decomposition of MFAPP and are then converted to pyrophosphate and polyphosphate species with viscous natures [47]. These phosphorus-containing acids react with the decomposed epoxy matrix through dehydration and esterification, promoting char formation [48]. Moreover, the viscous pyrophosphate and polyphosphate species tightly cover the surface of residual char (see Figure 5). These factors lead to the formation of a compact and phosphorus-rich char layer with polyaromatic/heteroaromatic structures, which acts as a physical barrier to isolate the underneath matrix from heat and oxygen. For EPV/MFAPP composites, the presence of disulfide bonds in the crosslinking networks leads to the degradation of the EPV composite at a lower temperature as presented in the TGA. They fracture during combustion to form sulfur-containing gases [45], which are released into the gas phase together with the decomposition gases of MFAPP, resulting in the formation of numerous pores on the surface of the char. The porous surface char layer cannot insulate the transfer of heat and oxygen, exhibiting relatively poor flame retardancy. 

## 4. Conclusions

This work performed a comparative study on the flame-retardant properties of EP/MFAPP and EPV/MFAPP composites to evaluate the flame-retardant potential of MFAPP in epoxy vitrimers. The results showed that the addition of 7.5 wt.% MFAPP endowed EP with excellent fire performance, including that the LOI value was as high as 29.9% and a V-0 rating was achieved in the UL-94 test (3.2 mm). Unfortunately, with the same loading (7.5 wt.%), although EPV/MFAPP7.5% showed an obvious anti-dripping performance, it did not reach any rating in the UL-94 test. SEM-EDS, XPS, and Raman were employed to assess the flame-retardant mechanism in the condensed phase. The results showed that the residue of EPV/MFAPP7.5% presented numerous holes during burning, which failed to form a continuous and dense char layer as a physical barrier resulting in relatively poor flame retardancy compared to EP/MFAPP7.5%. In summary, these results indicate that the disulfide-based epoxy vitrimers showed relatively high flammability, and it was difficult to improve their flame retardancy by adding small amounts of additive flame retardants (MFAPP). This study provides a perspective on the application of additive flame retardants in epoxy vitrimers and can contribute to future research on the improvement of flame retardancy in epoxy vitrimers.

## Figures and Tables

**Table 1:** Formulation of EP and EPV composites.

Sample	Composition				
	EP (g)	DDM (g)	DTDA (g)	MFAPP (g)	FRs (%)
EP	100	25.3	/	0	0
EP/MFAPP2.5%	100	25.3	/	3.21	2.5
EP/MFAPP5.0%	100	25.3	/	6.59	5
EP/MFAPP7.5%	100	25.3	/	10.16	7.5
EP/MFAPP10%	100	25.3	/	13.92	10
EPV	100	/	31.6	0	0
EPV/MFAPP2.5%	100	/	31.6	3.37	2.5
EPV/MFAPP5.0%	100	/	31.6	6.92	5
EPV/MFAPP7.5%	100	/	31.6	10.67	7.5
EPV/MFAPP10.0%	100	/	31.6	14.62	10

**Table 2:** TGA data from EP and EPV composites.

Sample	T_5%_ (°C)	T_50%_ (°C)	T_Max_ (°C)	Residues at 800 °C (wt%)
EP	375	406	388	19.7
EP/MFAPP2.5%	346	391	369	26.7
EP/MFAPP5.0%	338	385	355	28.6
EP/MFAPP7.5%	340	387	360	28.1
EP/MFAPP10.0%	337	380	354	29.1
EPV	308	367	350	15.5
EPV/MFAPP2.5%	306	359	339	21.3
EPV/MFAPP5.0%	304	351	328	24.6
EPV/MFAPP7.5%	303	353	324	27.8
EPV/MFAPP10.0%	305	352	324	27.6

**Table 3:** Related data from EP and EPV composites from LOI and UL-94.

Sample	LOI (%)	UL-94 Test			
		t_1_ (s)	t_2_ (s)	Dripping	Rating
EP	23.4	206.2	/	Yes	NR
EP/MFAPP2.5%	23.6	149.8	/	No	NR
EP/MFAPP5.0%	26.3	57.6	1.4	No	NR
EP/MFAPP7.5%	29.9	2.8	0.2	No	V-0
EP/MFAPP10%	35.5	0.2	0	No	V-0
EPV	19.9	165.8	/	Yes	NR
EPV/MFAPP2.5%	22.2	151.4	/	No	NR
EPV/MFAPP5.0%	23.1	149.6	/	No	NR
EPV/MFAPP7.5%	24.3	136.4	/	No	NR
EPV/MFAPP10.0%	25.3	142.2	/	No	NR

t_1_ and t_2_ refers to the average times of first and second ignitions; NR: no rating.

## Data Availability

The raw/processed data required to reproduce these findings cannot be shared at this time as the data also forms part of an ongoing study.
